# The clinical characteristics of pediatric patients infected by SARS-CoV-2 Omicron variant and whole viral genome sequencing analysis

**DOI:** 10.1371/journal.pone.0282389

**Published:** 2023-03-10

**Authors:** Hin Fung Tsang, Allen Chi Shing Yu, Aldrin Kay Yuen Yim, Nana Jin, Yu On Wu, Hennie Yuk Lin Cheng, WL Cheung, Wai Ming Stanley Leung, Ka Wai Lam, Tin Nok Hung, Loiston Chan, Jiachi Chiou, Xiao Meng Pei, On Ying Angela Lee, William Chi Shing Cho, Sze Chuen Cesar Wong

**Affiliations:** 1 Department of Clinical Laboratory and Pathology, Hong Kong Adventist Hospital, Hong Kong, China; 2 Department of Health Technology and Informatics, The Hong Kong Polytechnic University, Hong Kong, China; 3 Codex Genetics Limited, Hong Kong, China; 4 Department of Applied Biology & Chemical Technology, The Hong Kong Polytechnic University, Hong Kong, China; 5 Department of Clinical Oncology, Queen Elizabeth Hospital, Hong Kong, China; Imam Abdulrahman Bin Faisal University, SAUDI ARABIA

## Abstract

Pediatric population was generally less affected clinically by SARS-CoV-2 infection. Few pediatric cases of COVID-19 have been reported compared to those reported in infected adults. However, a rapid increase in the hospitalization rate of SARS-CoV-2 infected pediatric patients was observed during Omicron variant dominated COVID-19 outbreak. In this study, we analyzed the B.1.1.529 (Omicron) genome sequences collected from pediatric patients by whole viral genome amplicon sequencing using Illumina next generation sequencing platform, followed by phylogenetic analysis. The demographic, epidemiologic and clinical data of these pediatric patients are also reported in this study. Fever, cough, running nose, sore throat and vomiting were the more commonly reported symptoms in children infected by Omicron variant. A novel frameshift mutation was found in the ORF1b region (NSP12) of the genome of Omicron variant. Seven mutations were identified in the target regions of the WHO listed SARS-CoV-2 primers and probes. On protein level, eighty-three amino acid substitutions and fifteen amino acid deletions were identified. Our results indicate that asymptomatic infection and transmission among children infected by Omicron subvariants BA.2.2 and BA.2.10.1 are not common. Omicron may have different pathogenesis in pediatric population.

## Introduction

Coronavirus disease 2019 (COVID-19) is a viral respiratory disease caused by a novel coronavirus, severe acute respiratory syndrome coronavirus 2 (SARS-CoV-2) [1,2]. Pediatric population was generally less affected clinically by SARS-CoV-2 infection. Few pediatric cases of COVID-19 have been reported compared to those reported in infected adults. This discrepancy may be due to various reasons like the fact that most infected children are asymptomatic or the symptoms are too mild to draw medical attention [3]; the differences in various immune responses and physiological differences such as lower angiotensin-converting enzyme 2 (ACE2) expression in children relative to adults [4]; elevated baseline IgM targeting coronavirus antigens [5] and stronger early innate antiviral immune responses in children [6].

However, during the fifth COVID-19 outbreak in Hong Kong which was dominated by Omicron variant (B.1.1.529) between January 2022 and May 2022, there was a rapid increase in the hospitalization rate in SARS-CoV-2 infected pediatric patients [7]. Four deaths (0.35%) occurred among children aged between 0 and 11 years old [7]. The pediatric intensive care unit (PICU) admission rate during the Omicron variant-dominated outbreak was found to be 1.83%, which was higher than the total PICU admission rate in the previous outbreaks caused by other variants (0.14%) [7] Neurological complications occurred in 14.91% of hospitalized children [7]. The Omicron variant was first identified in South Africa in early November 2021 and classified as a variant of concern (VOC) by the World Health Organization (WHO) [7,8]. Because of its high transmission rate as well as its remarkable ability of immune escape, this variant quickly became the dominant variant across multiple countries and regions and sparked the fifth wave of COVID-19 outbreak in Hong Kong [7].

SARS-CoV-2 infection may cause mild signs and symptoms but can also affect multiple organs simultaneously as organs and cells harboring ACE-2 receptors are the primary targets of SARS-CoV-2. Recent studies have highlighted the effects of COVID-19 on multiple organs and systems, including respiratory, cardiovascular, gastrointestinal, urinary, nervous, endocrine, reproductive, immune and integumentary systems [9]. To understand the epidemiology, infection risk, severity and viral genome of SARS-CoV-2 in children, a total of sixty-eight SARS-CoV-2 genome sequences isolated from infected pediatric patients in Hong Kong during the fifth wave of COVID-19 outbreak were analyzed by whole viral genome amplicon sequencing on Illumina platform, followed by phylogenetic and phylodynamic analysis. The demographic, epidemiologic and clinical data of these pediatric patients are also reported in this study Of the sixty-eight genome sequences, sixty-five (95.6%) generated analyzable results. All of the analyzable strains belonged to the PANGO lineage B.1.1.529 as classified by Pangolin (version 4.0.2) and Nextclade clade 21L (version 1.11.0), Omicron variant.

## Patients and methods

### Specimen collection

Nasopharyngeal swab (NPS), combined nasal and throat swab (CNTS) and posterior oropharyngeal saliva (POS) were collected from sixty-eight RT-PCR confirmed SARS-CoV-2 infected pediatric patients (fifty-five NPS, six CNTS and seven POS) aged between 0 and 17 years old between January 2022 and March 2022 at Hong Kong Adventist Hospital in Hong Kong. All SARS-CoV-2 RT-PCR results were confirmed using both cobas^®^ Liat^®^ SARS-CoV-2 & Influenza A/B assay (Roche Molecular Systems, Inc., Pleasanton, CA) and cepheid^®^ Xpress SARS-CoV-2 assay (Cepheid, Sunnyvale, CA) as described in our previous study [10]. NPS and CNTS samples were collected in viral transport medium (VTM). As for POS, patients were instructed to expectorate saliva into a sterile container. No food or drink, mouthwash and brushing teeth within two hours before the specimen collection [9]. This study was approved by the institutional review board of the Hong Kong Adventist Hospital (HKAH2021001). Written informed consent was waived since archived NPS, CNTS and POS were used.

### Whole viral genome sequencing

#### Viral RNA extraction

Viral RNA was extracted from NPS, CNTS and POS specimens using QIAamp^®^ Viral RNA Mini Kit (Qiagen, Germany) according to manufacturer’s instruction.

#### Illumina whole genome amplicon sequencing

Extracted RNA was first reverse transcribed to cDNA and subjected to whole-genome targeted amplification of SARS-CoV-2 sequences using COVIDSeq^TM^ SARS-CoV-2 Primer Panel (Illumina, USA), which contain primer sequences defined by the ARTIC network (ARTIC v4.1). The resulting ~400bp amplicons were fragmented, end repaired, and A-tailed using COVIDSeq^TM^ Library Prep Kit (Illumina, USA) according to manufacturer’s instruction. Unique dual indexes were added to the samples using the 10 base pair index i7 and i5 adapters of COVIDSeq^TM^ (Illumina, USA) according to manufacturer’s instruction. The size distributions of the sequencing libraries were checked using Tapestation 4150 (Agilent, USA). KAPA Pure Beads were used to perform double size selection of the libraries and equimolar pooling of libraries was performed by quantifying the samples using Qubit 4 fluorometer (Thermo Fisher, USA). Libraries were sequenced using NextSeq2000 P2 2 x 100bp kit (Illumina, USA) with at least 10M reads per sample.

#### Bioinformatic analysis

Sequencing data was analyzed using DRAGEN COVID Lineage (version 3.5.9; Illumina Inc., USA) and method as described previously [11,12]. In brief, sequencing reads of human origin were removed using NCBI Human Read Scrubber algorithm. ARTIC primer sequences were removed from the reads, followed by aligning to the reference genome Wuhan-Hu-1 (GenBank accession number MN908947.3) using DRAGEN (Illumina Inc., USA). Samples with less than 90 amplicons detected are filtered. Variant calling and consensus genome assembly with respect to the reference genome were performed using DRAGEN (Illumina Inc., USA) using default parameters. Nucleotide and amino acid positions were numbered according to the reference genome. All genome sequences analyzed in this study were submitted to GISAID under accession numbers EPI_ISI_13822514 to 13822565 and EPI_ISI_14336314 to 14336324. Pangolin lineages of the consensus genomes were assigned using Pangolin COVID-19 Lineage Assigner (version 4.0.2) [13]. Phylogenetic clades of the consensus genomes were mapped using Nextclade (version 1.11.0) [14].

## Results

**Fig 1:**
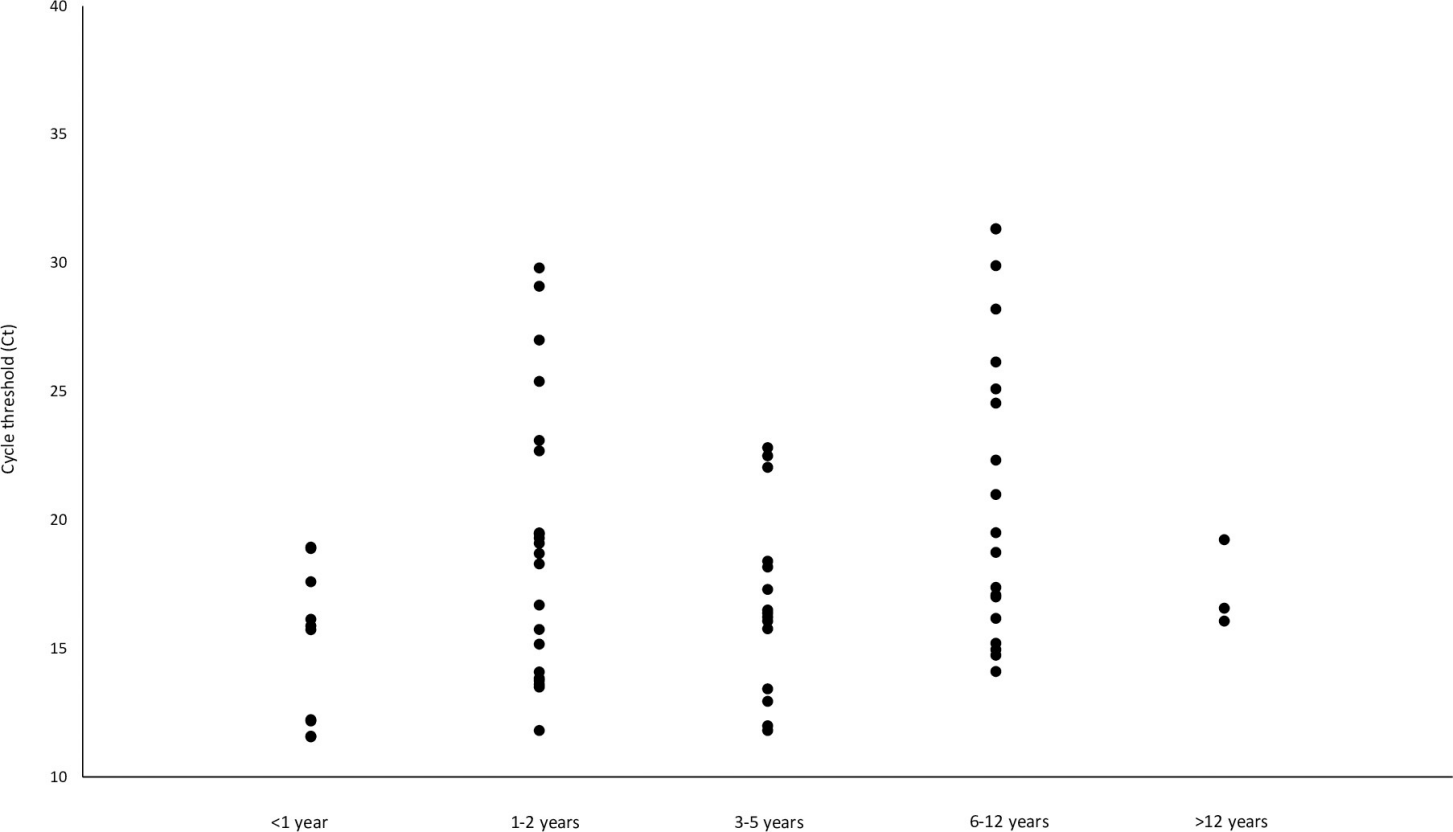
Cycle threshold (Ct) values of the 68 RT-PCR confirmed SARS-CoV-2 infected pediatric patients aged between 0 and 17 years old (obtained by cobas^®^ Liat^®^ SARS-CoV-2 & Influenza A/B assay).

A total of sixty-eight laboratory-confirmed SARS-CoV-2 infected children aged between 0 and 17 years old were studied (mean Ct value: 18.5 ± 5.2 obtained by cobas^®^ Liat^®^ SARS-CoV-2 & Influenza A/B assay) **(Fig 1)**. Among these cases, ten cases (14.7%) were in children aged below 1 year old, twenty-one cases (30.9%) were in children aged between 1 and 2 years old, fifteen cases (22.1%) were in children aged between 3 and 5 years old, ninteen cases (27.9%) were in children aged between 6 and 12 years old and three cases (4.4%) were in children aged between 13 and 17 years of age (**Table 1**).

**Table 1:** Clinical characteristics and demographics of the SARS-CoV-2 infected pediatric patients (n = 68) aged between 0 and 17 years.

**Age**			<1 years	1–2 years	3–5 years	6–12 years	>12 years	Total
			(n = 10)	(n = 21)	(n = 15)	(n = 19)	(n = 3)	(n = 68)
**Sex**		Male	7 (70%)	12 (57.1%)	9 (60%)	11 (57.9%)	3 (100%)	42 (61.8%)
		Female	3 (30%)	9 (42.9%)	6 (40%)	8 (42.1%)	0 (0%)	26 (38.2%)
**Signs and Symptoms**		Fever	10 (100%)	20 (95.2%)	14 (93.3%)	17 (89.5%)	3 (100%)	64 (94.1%)
		Cough	3 (30%)	6 (28.6%)	5 (33.3%)	6 (31.6%)	3 (100%)	23 (33.8%)
		Sore throat	N/A	N/A	3 (20%)	7 (36.8%)	2 (66.7%)	12^ (32.4%)
		Running nose	1 (10%)	10 (47.6%)	5 (33.3%)	6 (31.6%)	1 (33.3%)	23 (33.8%)
		Diarrhea	2 (20%)	0 (0%)	0 (0%)	0 (0%)	0 (0%)	2 (2.9%)
		Short of breath	0 (0%)	1 (4.8%)	0 (0%)	0 (0%)	0 (0%)	1 (1.5%)
		Vomiting	1 (10%)	8 (38.1%)	2 (13.3%)	5 (26.3%)	0 (0%)	16 (23.5%)
		Oxygen saturation < 94%	0 (0%)	0 (0%)	0 (0%)	0 (0%)	0 (0%)	0 (0%)
		Increased heart rate#	6 (60%)	10 (47.6%)	3 (20%)	5 (26.3%)	2 (66.7%)	26 (38.2%)
		Increased respiratory rate*	1 (10%)	0 (0%)	0 (0%)	0 (0%)	0 (0%)	1 (1.5%)
		With 1 sign or symptom	3 (30%)	5 (23.8%)	4 (26.7%)	5 (26.3%)	0 (0%)	17 (25%)
		More than 1 sign or symptom	7 (70%)	16 (76.2%)	11 (73.3%)	14 (73.7%)	3 (100%)	51 (75%)
		No sign or symptom	0 (0%)	0 (0%)	0 (0%)	0 (0%)	0 (0%)	0 (0%)
**Co-infection**	Influenza A		0 (0%)	0 (0%)	0 (0%)	0 (0%)	0 (0%)	0 (0%)
	Influenza B		0 (0%)	0 (0%)	0 (0%)	0 (0%)	0 (0%)	0 (0%)
						
**Travel History**	With travel history outside Hong Kong in past 21 days		0 (0%)	0 (0%)	0 (0%)	0 (0%)	0 (0%)	0 (0%)
**Contact history**	Contacted with suspected / confirmed COVID-19 cases in past 21 days		0 (0%)	1 (4.7%)	0 (0%)	1 (5.2%)	0 (0%)	2 (2.9%)
**Cluster**	Cohabited family members shared similar symptoms		0 (0%)	3 (14.3%)	1 (6.7%)	2 (10.5%)	1 (33.3%)	9 (13.2%)

Remarks

# <1year: >160/min, 1-2years: >150/min, 3-5years: >140/min, 6-12years: >120/min, >12years: >100/min.

* <1year: >40/min, 1-2years: >35/min, 3-5years: >30/min, 6-12years: >25/min, >12years: >20/min.

^ patients aged between 3 and 17 years old.

### Clinical presentation

**Fig 2:**
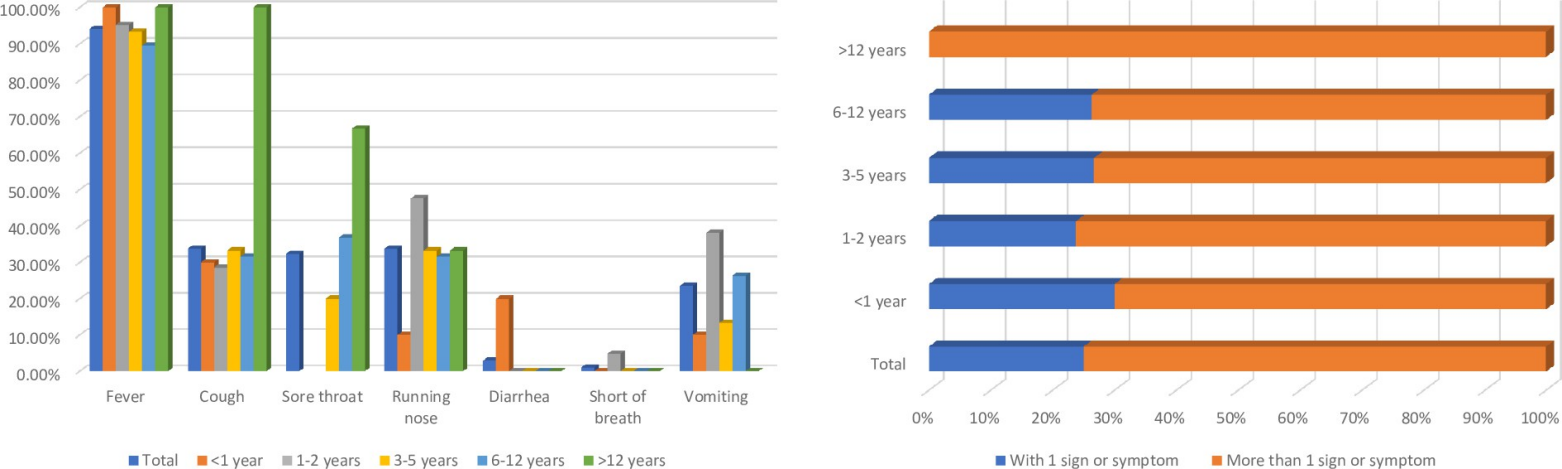
Signs and symptoms of the children infected by Omicron subvariants BA.2.2 and BA.2.10.1. (Right) Fifty-one children (75%) present more than 1 sign or symptom while 17 children (25%) present 1 sign or symptom only. (Left) The most common symptoms reported were fever (94.1%), cough (33.8%), running nose (33.8%), sore throat (32.4%) and vomiting (23.5%). Two children (2.9%) had diarrhea and 1 child (1.5%) had short of breath.

**Fig 3:**
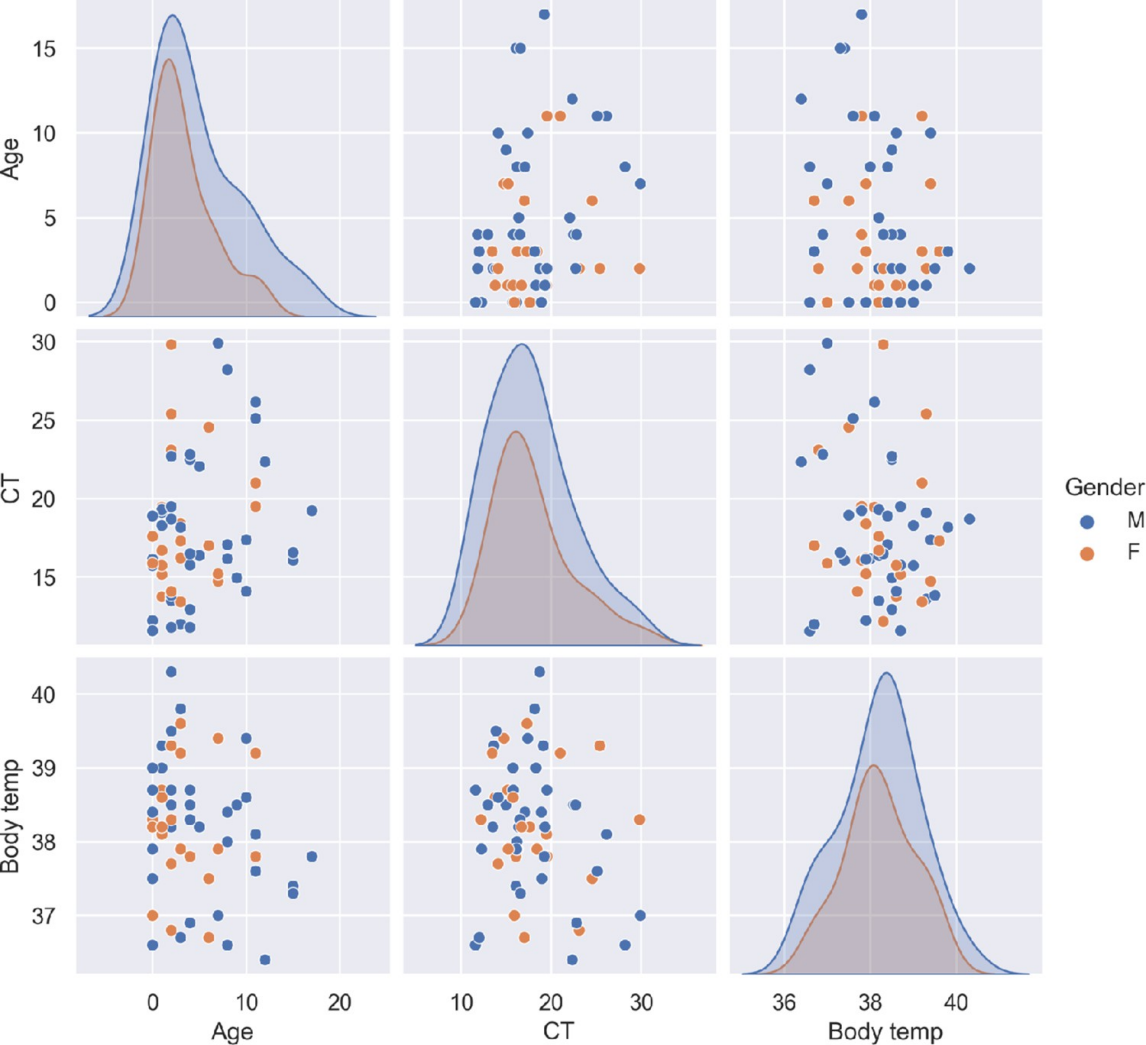
Distribution of CT values, body temperature and age of pediatric patients infected by Omicron variants BA.2.10.1 and BA.2.2. No sexual dimorphism was observed in this study.

Results of this study suggest a milder course of disease in children infected by Omicron subvariants BA.2.2 and BA.2.10.1. However, no asymptomatic case was identified. Fifty-one children (75%) presented more than one sign or symptom while seventeen children (25%) presented one sign or symptom only. No case of co-infection was found. The most common symptoms reported were fever (94.1%), cough (33.8%), running nose (33.8%), sore throat (32.4%) and vomiting (23.5%). Two children (2.9%) had diarrhea and one child (1.5%) had shortness of breath (**Table 1, Fig 2**). Children in this cohort infected by Omicron subvariants BA.2.2 and BA.2.10.1 showed similar signs and symptoms of infections. There was no evidence to show that children infected by Omicron subvariant BA.2.10.1 would result in worse and more severe clinical outcomes than children infected by BA.2.2. No sexual dimorphism was observed (**Fig 3**). Among all the 68 pediatric cases of SARS-CoV-2 infection, none of them displayed any underlying conditions.

### Transmission

In nine cases (13.2%), cohabiting family members were reported to have similar symptoms as the infected children. Two cases (2.9%) were reported to have a contact history with suspected or confirmed COVID-19 cases in the past 21 days. None of the cases had travel history outside Hong Kong in the past 21 days. This indicates that all the sixty-eight pediatric COVID-19 cases were local infections.

### Phylogenetic analysis

**Fig 4:**
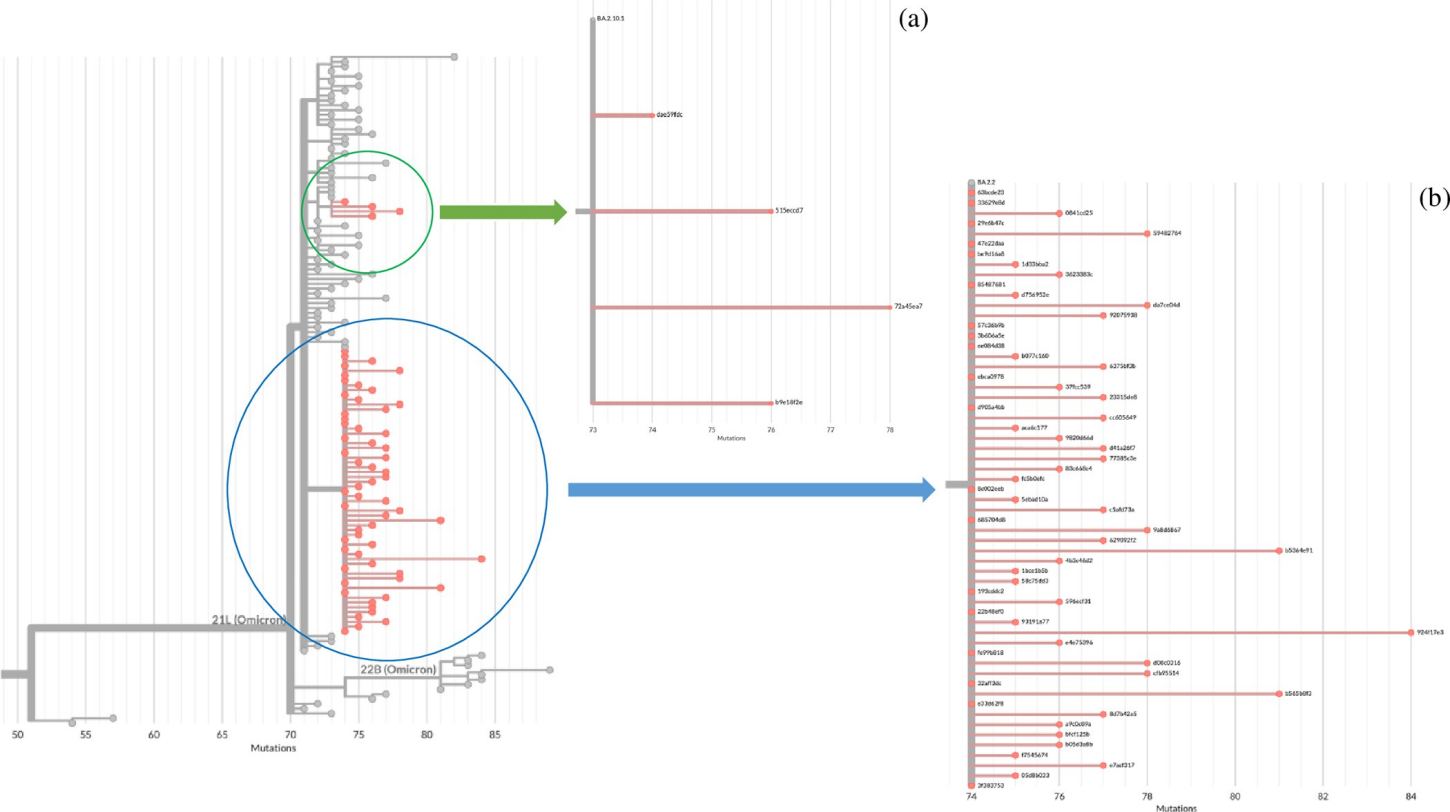
The maximum-likelihood phylogenetic tree of the 65 SARS-CoV-2 genome sequences isolated from pediatric patients between January 2022 and March 2022, during the fifth wave of COVID-19 outbreak in Hong Kong. The phylogenetic tree was rooted on the Wuhan-Hu-1 reference genome sequence and was inferred using IQ-Tree (version 2.1.2) and the GTR+G substitution model and 1000 bootstrap replicates using UFBoot. Tree visualization was generated using FigTree. All of the strains belonged to the PANGO lineage B.1.1.529 (b) and Nextclade clade 21L (a), Omicron variant. Two distinctive groups consisted of a group of 4 genome sequences that belong to subvariant BA.2.10.1 and a second group consisted of 61 genome sequences belonging to subvariant BA.2.2 were identified.

Two distinctive groups were identified. The first group consisted of four genome sequences that belong to subvariant BA.2.10.1 whereas the second group consisted of sixty-one genome sequences that belong to subvariant BA.2.2 (**Fig 4**).

### Viral genome mutations analysis

Compared to the reference genome of Wuhan-Hu-1 strain, a total of one nucleotide insertion, one frameshift mutation, one hundred and eighteen nucleotide substitutions, four nucleotide deletions and seven PCR primers and probes changes were identified. On protein level, eighty-three amino acid substitutions and fifteen amino acid deletions were identified in the B.1.1.529 Omicron variant (**Table 2**).

**Table 2:** Whole viral genome sequencing statistic and features of B.1.1.529 Omicron variant. All genome sequences analyzed in this study were submitted to GISAID under accession numbers EPI_ISI_13822514 to 13822565 and EPI_ISI_14336314 to 14336324.

Sequence ID	Sequence length (bp)	Genomic coverage > = 30x (%)	Total insertions	Frameshift mutations	No. of amino acid substitutions	No. of amino acid deletions	Total PCR primers change	Nextstrain clade	PANGO Sub-lineage
1bce1b5b	29,903	98.96	0	0	54	12	5	21L	BA.2.2
bfcf125b	29,903	99.02	0	0	53	12	5	21L	BA.2.2
58c75dd3	29,903	98.94	0	0	51	12	5	21L	BA.2.2
32aff3dc	29,903	99.70	0	0	53	12	5	21L	BA.2.2
3f383753	29,903	99.70	0	0	53	12	5	21L	BA.2.2
ebca0978	29,903	99.51	0	0	53	12	5	21L	BA.2.2
22b48ef0	29,903	86.06	0	0	51	12	5	21L	BA.2.2
4b3c46d2	29,903	96.54	0	0	52	12	5	21L	BA.2.2
d756952e	29,903	99.59	0	0	52	12	5	21L	BA.2.2
6375bf3b	29,903	99.31	0	0	53	12	5	21L	BA.2.2
72a45ea7	29,903	96.95	1	1	49	12	5	21L	BA.2.10.1
b565b8f3	29,903	97.35	1	1	51	12	5	21L	BA.2.2
83c668c4	29,903	98.99	1	1	49	12	5	21L	BA.2.2
685704d8	29,903	99.00	0	0	52	12	5	21L	BA.2.2
59482764	29,903	97.42	1	1	51	12	5	21L	BA.2.2
9820d66d	29,903	98.97	0	0	52	12	5	21L	BA.2.2
63bcde23	29,903	99.01	0	0	53	12	5	21L	BA.2.2
33629e8d	29,903	99.59	0	0	53	12	5	21L	BA.2.2
8c002eeb	29,903	99.02	0	0	53	12	5	21L	BA.2.2
c5afd73a	29,903	98.10	1	1	50	12	5	21L	BA.2.2
93191a77	29,903	99.70	0	0	53	12	5	21L	BA.2.2
d905a4bb	29,903	98.90	0	0	53	12	5	21L	BA.2.2
be9d16a8	29,903	98.96	1	1	50	12	5	21L	BA.2.2
515eccd7	29,903	99.02	0	0	53	12	5	21L	BA.2.10.1
b9e18f2e	29,903	99.52	0	0	55	12	5	21L	BA.2.10.1
77385c3e	29,903	98.97	1	1	50	12	5	21L	BA.2.2
85487681	29,903	99.02	0	0	53	12	5	21L	BA.2.2
dae59fdc	29,903	99.70	0	0	53	12	5	21L	BA.2.10.1
29e6b47c	29,903	83.21	0	0	53	12	5	21L	BA.2.2
0841cd25	29,903	98.99	0	0	51	12	5	21L	BA.2.2
47e22daa	29,903	99.69	0	0	53	12	5	21L	BA.2.2
9a8d6867	29,903	98.91	1	1	50	12	5	21L	BA.2.2
ee084d38	29,903	88.12	0	0	51	12	5	21L	BA.2.2
193cddc2	29,903	99.01	0	0	53	12	5	21L	BA.2.2
fe99b818	29,903	99.55	0	0	53	12	5	21L	BA.2.2
aca6c177	29,903	88.43	0	0	51	12	5	21L	BA.2.2
3b606a5e	29,903	98.86	1	1	50	12	5	21L	BA.2.2
f7545674	29,903	93.08	0	0	52	12	5	21L	BA.2.2
23315de8	29,903	99.03	0	0	53	12	5	21L	BA.2.2
92075938	29,903	99.00	0	0	52	12	5	21L	BA.2.2
3623383c	29,903	99.02	0	0	53	12	5	21L	BA.2.2
cfb95514	29,903	74.98	0	0	47	12	5	21L	BA.2.2
05d8b033	29,903	99.70	0	0	54	12	5	21L	BA.2.2
1d33bba2	29,903	98.89	0	0	52	12	5	21L	BA.2.2
b05d3a8b	29,903	99.71	0	0	53	12	5	21L	BA.2.2
924f17e3	29,903	90.14	0	1	53	13	6	21L	BA.2.2
da7ce04d	29,903	99.66	0	0	53	12	5	21L	BA.2.2
e33d62f8	29,903	99.01	0	0	52	12	5	21L	BA.2.2
629092f2	29,903	99.69	0	0	54	12	5	21L	BA.2.2
d41a26f7	29,903	92.62	0	0	51	12	5	21L	BA.2.2
5ebad10a	29,903	99.70	0	0	53	12	5	21L	BA.2.2
e7acf317	29,903	99.70	0	0	54	12	5	21L	BA.2.2
b077c160	29,903	98.97	0	0	53	12	5	21L	BA.2.2
fc5b0efc	29,903	99.70	0	0	53	12	5	21L	BA.2.2
a9c0c89a	29,903	99.70	0	0	53	12	5	21L	BA.2.2
57c36b9b	29,903	99.70	0	0	53	12	5	21L	BA.2.2
8d7b42a5	29,903	99.69	0	0	53	12	5	21L	BA.2.2
e4e75396	29,903	98.80	0	0	53	12	5	21L	BA.2.2
b5364e91	29,903	79.07	0	0	47	12	5	21L	BA.2.2
37fcc539	29,903	99.69	0	0	54	12	5	21L	BA.2.2
596ecf31	29,903	99.70	0	0	55	12	6	21L	BA.2.2
d08c0316	29,903	98.97	0	0	54	12	5	21L	BA.2.2
cc605649	29,903	99.69	0	0	55	12	5	21L	BA.2.2

#### Nucleotide insertion

One nucleotide insertion (14760T) was found in nine sequenced subjects at the non-structural protein 12 (NSP12) of the open reading frame 1b (ORF1b), leading to a novel frameshift mutation.

#### Nucleotide substitutions

A total of one hundred and eighteen nucleotide substitutions were identified in this cohort (**Table 3**). Forty (33.9%) of them were found in ORF1a (C222T, C241T, C583T, T670G, C823T, C1469T, C2433T, G2527A, C2790T, C2841T, T2992C, C3037T, G4184A, C4321T, G4371A, C4423T, T4591C, C4893T, C5259T, A5914G, C8441A, C8991T, G9136A, C9344T, A9424G, C9491T, C9534T, C9866T, C10029T, C10198T, G10447A, C10449A, T10696C, G11115T, C12525T, G12753T, C12880T, C13072T, C13119T and C13255T); fourteen (11.9%) were found in ORF1b (C14408T, A14748C, A14753G, T15521A, C15714T, T16342C, G16526T, C17410T, T17694C, A18163G, C19955T, A20055G, G20580T and C20759T); thirty-six (30.5%) were found in spike (S) gene (C21618T, G21987A, A22125T, T22200G, G22577T, G22578A, C22674T, T22679C, C22686T, A22688G, G22775A, A22786C, G22813T, C22879T, T22882G, G22992A, C22995A, A23013C, A23040G, A23055G, A23063T, T23075C, A23403G, C23525T, T23599G, C23604A, C23854A, G23948T, G23955A, A24036G, A24424T, T24469A, C25000T, G25166C, T25224C and A25315C); three (2.5%) were found in ORF3a (C25584T, C26060T and C26198T); one (0.8%) was found in envelope (E) gene (C26270T); five (4.2%) were found in membrane (M) gene (C26577G, G26709A, C26858T, C26912T and A27006C); four (3.4%) were found in ORF6 (A27259C, G27382C, A27383T and T27384C); one (0.8%) was found in ORF7a (C27741T); one (0.8%) was found in ORF7b (C27807T); one (0.8%) was found in ORF9b (G28436T) and twelve (10.2%) were found in nucleocapsid (N) gene (A28271T, C28311T, C28606T, C28770T, G28881A, G28882A, G28883C, T29404C, A29436G, A29510C, C29640T and G29706T). Of these changes, eighty-three (70.3%) of them resulted in nonsynonymous mutations, while thirty-five (29.7%) of them resulted in synonymous mutations.

**Table 3:** Nucleotide and nonsynonymous amino acids mutations identified in the genome sequences of SARS-CoV-2 B.1.1.529 Omicron subvariants BA.2.2 and BA.2.10.1.

Affected genes	Nucleotide position	Wuhan-Hu-1 MN908947	BA.2.2 (Proportion in this study)	BA.2.10.1 (Proportion in this study)	Amino acid changes	Amino acid deletion
ORF1a	222	C	T (1.7%)	C	N/A	S3675-, G3676-, F3677-, V3689-
ORF1a	241	C	T (84.5%)	T (100%)	N/A	
					
ORF1a	670	T	G (100%)	G (100%)	S135R	
					
ORF1a	1,469	C	T (1.7%)	C	R402C	
ORF1a	2,433	C	T (1.7%)	C	S723F	
					
ORF1a	2,790	C	T (100%)	T (100%)	T842I	
ORF1a	2,841	C	C	T (100%)	A859V	
					
					
ORF1a	4,184	G	A (100%)	A (100%)	G1307S	
					
ORF1a	4,371	G	A (1.7%)	G	G1369E	
					
					
ORF1a	4,893	C	T (100%)	C	T1543I	
ORF1a	5,259	C	T (1.7%)	C	T1665I	
					
					
ORF1a	8,991	C	T (1.7%)	C	A2909V	
ORF1a	9,136	G	A (1.7%)	G	M2957I	
ORF1a	9,344	C	T (96.6%)	T (100%)	L3027F	
					
ORF1a	9,491	C	T (1.7%)	C	H3076Y	
ORF1a	9,534	C	T (100%)	T (100%)	T3090I	
ORF1a	9,866	C	T (100%)	T (100%)	L3201F	
ORF1a	10,029	C	T (100%)	T (100%)	T3255I	
					
					
ORF1a	10,449	C	A (100%)	A (100%)	P3395H	
					
ORF1a	11,115	G	T (1.7%)	G	G3617V	
ORF1a	12,525	C	T (100%)	C	T4087I	
ORF1a	12,753	G	T (1.7%)	G	C4163F	
ORF1a	12,880	C	T (100%)	T (100%)	I4205I	
					
ORF1a	13,119	C	T (1.7%)	C	A4285V	
					
ORF1b	14,408	C	T (100%)	T (100%)	P314L	P403-, G404-
ORF1b	14,748	A	A	C (50%)	E4828D	
ORF1b	14,753	A	G (3.4%)	G (50%)	K4830R	
ORF1b	15,521	T	A (1.7%)	T	F5086V	
					
ORF1b	16,342	T	T	C (75%)	S5360P	
ORF1b	16,526	G	T (1.7%)	G	C5421F	
ORF1b	17,410	C	T (100%)	T (100%)	R5716C	
					
ORF1b	18,163	A	G (84.5%)	G (100%)	I1566V	
ORF1b	19,955	C	T (58.6%)	T (50%)	T6564I	
					
					
ORF1b	20,759	C	T (1.7%)	C	A6832V	
S	21,618	C	T (96.6%)	T (100%)	T19I	L24-, P25-, P26-
S	21,987	G	A (100%)	A (100%)	G142D	
S	22,125	A	A	T (50%)	N188I	
S	22,200	T	G (100%)	G (100%)	V213G	
S	22,577	G	T (1.7%)	G	G339C	
S	22,578	G	A (100%)	A (100%)	G339D	
S	22,674	C	T (98.3%)	T (100%)	S371F	
S	22,679	T	C (96.6%)	C (100%)	S373P	
S	22,686	C	T (96.6%)	T (100%)	S375F	
S	22,688	A	G (96.6%)	G (100%)	T376A	
S	22,775	G	A (98.3%)	A (100%)	D405N	
S	22,786	A	C (87.9%)	C (100%)	R408S	
S	22,813	G	T (100%)	T (100%)	K417N	
					
S	22,882	T	G (100%)	G (100%)	N440K	
S	22,992	G	A (100%)	A (100%)	S477N	
S	22,995	C	A (100%)	A (100%)	T478K	
S	23,013	A	C (100%)	C (100%)	E484A	
S	23,040	A	G (100%)	G (100%)	Q493R	
S	23,055	A	G (100%)	G (100%)	Q498R	
S	23,063	A	T (100%)	T (100%)	N501Y	
S	23,075	T	C (100%)	C (100%)	Y505H	
S	23,403	A	G (100%)	G (100%)	D614G	
S	23,525	C	T (100%)	T (100%)	H655Y	
S	23,599	T	G (100%)	G (100%)	N679K	
S	23,604	C	A (100%)	A (100%)	P681H	
S	23,854	C	A (100%)	A (100%)	N764K	
S	23,948	G	T (100%)	T (100%)	D796Y	
S	23,955	G	G	A (100%)	G798D	
S	24,036	A	A	G (50%)	K825R	
S	24,424	A	T (100%)	T (100%)	Q954H	
S	24,469	T	A (100%)	A (100%)	N969K	
					
S	25,166	G	C (1.7%)	G	E1202Q	
S	25,224	T	C (98.3%)	T	I1221T	
					
						N/A
ORF3a	26,060	C	T (100%)	T (100%)	T233I	
ORF3a	26,198	C	T (1.7%)	C	T269M	
E	26,270	C	T (100%)	T (100%)	T9I	N/A
M	26,577	C	G (100%)	G (100%)	Q19E	N/A
M	26,709	G	A (82.8%)	A (75%)	A63T	
					
					
M	27,006	A	C (1.7%)	C	K162Q	
						N/A
ORF6	27,382	G	C (100%)	C (100%)	D61L	
ORF6	27,383	A	T (100%)	T (100%)	D61L	
ORF6	27,384	T	C (100%)	C (100%)	D61L	
						N/A
						N/A
N	28,271	A	T (100%)	T (100%)	N/A	N/A
N	28,311	C	T (100%)	T (100%)	P13L	
ORF9b	28,436	G	T (6.9%)	G	A55S	E27-, N28-, A29-
						E31-, R32-, S33-
N	28,770	C	T (1.7%)	C	T166I	
N	28,881	G	A (100%)	A (100%)	R203K	
N	28,882	G	A (100%)	A (100%)	R203K	
N	28,883	G	C (100%)	C (100%)	G204R	
					
N	29,436	A	G (1.7%)	A	K388R	
N	29,510	A	C (98.3%)	C (100%)	S413R	
N	29,640	C	T (1.7%)	C	A28V	
N	29,706	G	T (1.7%)	G	N/A	

ORF: Open reading frame, S: Spike, E: Envelop, M: Membrane, N: Nucleocapsid.

#### Nucleotide deletions

A total of four nucleotide deletions were identified in ORF1a (between positions 11288 and 11296) (100%), ORF1b (between positions 21633 and 21641) (100%), ORF9b (between positions 28362 and 28370) (100%) and N gene (between positions 29734 and 29759) (93.8%).

#### Amino acid substitutions

A total of eighty-three amino acid substitutions were identified in this cohort (**Table 3**). One (1.2%) of the substitutions was found in E protein (T9I), three (3.6%) were found in M protein (mutational hotspots: Q19E and A63T), eight (9.6%) were found in N protein (mutational hotspots: P13L, R203K, G204R and S413R), twenty-one (25.3%) were found in ORF1a (mutational hotspots: S135R, T842I, G1307S, T1543I, L3201F, T3255I and P3395H), thirteen (15.7%) were found in ORF1b (mutational hotspots: P314L, R5716C and I1566V), two (2.4%) was found in ORF3a (mutational hotspot: T223I), one (1.2%) was found in ORF6 (D61L), one (1.2%) was found in ORF9b (P10S) and thirty-three (39.8%) were found in S protein (mutational hotspots: T19I, G142D, V213G, G339D, S371F, S373P, S375F, T376A, D405N, R408S, K417N, N440K, S477N, T478K, Q493R, Q498R, N501Y, Y505H, D614G, H655Y, N679K, P681H, N764K, D796Y, Q954H, N969K and I1221T).

#### Amino acid deletions

A total of fifteen amino acid deletions were identified in this cohort. Three (20%) of these deletions were found in N protein (E31-, R32- and S33-), four (26.7%) in ORF1a (S3675-, G3676-, F3677- and V3689-), two (13.3%) in ORF1b (P403- and G404-), three (20%) in ORF9b (E27-, N28- and A29-), three (20%) in S protein (L24-, P25- and P26-).

#### PCR primers and probes changes

A total of seven polymerase chain reaction (PCR) primers and probes changes in WHO listed SARS-CoV-2 primers and probes were found in Omicron subvariants BA.2.2 and BA.2.10.1 (**Table 4**). Of the seven changes, five (71.4%) were located in N gene. C28770T was located within the N gene probe of Charité, G28881A, G28882A and G28883C were located within the N gene forward primer of China CDC, whereas C28311T was located within the N1 gene probe of US CDC. One change (14.3%) was located in RdRp gene. T15521A was located within the RdRp gene reverse primer of Charité. One (14.3%) was located in E gene. On the other hand, C26270T was located within the E gene forward primer of Charité.

**Table 4:** The polymerase chain reaction (PCR) primers and probes changes in WHO listed SARS-CoV-2 primers and probes found in Omicron subvariants BA.2.2 and BA.2.10.1. The positions of the nucleotide changes in the primer/probe sequences are highlighted.

Gene targets	Primers/probes	Oligonucleotide sequences (5’-3’)	Changes identified
N gene	N gene forward primer of China CDC	GGG GAA CTT CTC CTG CTA GAA T	G28881A, G28882A, G28883C
N gene	N1 gene probe of US CDC	ACC CCG CAT TAC GTT TGG TGG ACC	C28311T
N gene	N gene probe of Charité, Germany	ACT TCC TCA AGG AAC AAC ATT GCC A	C28770T
RdRp gene	RdRp gene reverse primer of Charité, Germany	CAR ATG TTA AAW ACA CTA TTA GCA TA	T15521A
E gene	E gene forward primer of Charité, Germany	ACA GGT ACG TTA ATA GTT AAT AGC GT	C26270T

W: A/T; R: G/A.

## Discussion

To understand the epidemiology, infection risk, severity and viral genome of SARS-CoV-2 in children, a total of sixty-eight SARS-CoV-2 genome sequences collected from pediatric patients between January 2022 and May 2022 were determined by whole viral genome amplicon sequencing using Illumina next generation sequencing platform, followed by phylogenetic analysis. The more commonly presented symptoms in children infected by Omicron variant were fever, cough, running nose, sore throat and vomiting. Of the sixty-eight pediatric cases, fifty-one children (75%) presented more than one sign or symptom while seventeen children (25%) presented one sign or symptom only. No asymptomatic cases were identified. The cycle threshold (Ct) values in the sixty-eight pediatric cases were low (mean Ct value: 18.5 ± 5.2), showing that the viral load was high in these cases and these infected children were infectious to others. The data suggests that asymptomatic infection and transmission among children infected by Omicron subvariants BA.2.2 and BA.2.10.1 are not common. In Hong Kong, only children aged above 3 were recommended to receive COVID-19 vaccination. In this cohort, thirty-one children (45.6%) were aged below 3. Of the other thirty-seven children (54.4%) aged above 3, six (16.2%) of them have received COVID-19 vaccination. The pediatric population was generally less affected clinically by SARS-CoV-2 infection in the past few waves of COVID-19 outbreaks caused by variants other than Omicron. This discrepancy in clinical responses between adults and pediatric population may have been due to the differences in various immune responses and physiological differences such as lower ACE2 expression in children relative to adults [4], elevated baseline IgM targeting coronavirus antigens [5] and stronger early innate antiviral immune responses in children [6]. However, during this Omicron variant (B.1.1.529) dominated fifth wave of COVID-19 outbreak in Hong Kong, there was a rapid increase in the hospitalization rate in SARS-CoV-2 infected pediatric patients. In some rare cases (0.002%) of the pediatric cases, some children developed multisystem inflammatory syndrome (MIS-C) after being infected by the SARS-CoV-2 virus [15,16].

In a recent study that compared the antibody specificity in COVID-19 pediatric population to that in adults, the antibodies response against the structural protein E and accessory protein ORF8 was found to be significantly elevated in SARS-CoV-2 infected pediatric population, compared to adults [16]. In contrast, antibodies against structural proteins S1 and M and accessory proteins ORF3a and ORF7b were found to be reduced compared to adults [16]. From our whole viral genome analysis, no amino acid mutations were found in the accessory proteins ORF8 and ORF7b of the SARS-CoV-2 Omicron subvariants BA.2.2 and BA.2.10.1 in the infected pediatric population. Pediatric population might be more susceptible to SARS-CoV-2 Omicron variant ORF8 wildtype. SARS-CoV-2 ORF8 is a 121-amino acid long accessory protein that mainly acts as immune-modulator to down-regulate MHC class I molecules in order to shield the infected cells from cytotoxic T cells [16–18]. On the other hand, ORF8 is a potent inhibitor that suppresses type I interferon responses [17–19]. ORF8 modulates both adaptive host immunity and innate immune responses and studies have demonstrated that ORF8 is associated with the severity of COVID-19 [17,20]. One amino acid substitution (T9I) was found at the transmembrane region of E proteins [21]. However, no association between this mutation and the transmission and severity of COVID-19 could be identified. Three amino acid substitutions (Q19E, A63T and K162Q) were found in M protein, twenty-five amino acid substitutions (T19I, A27S, G142D, N188I, V213G, G339D, S371F, S373P, S375F, T376A, D405N, R408S, K417N, N440K, S477N, T478K, E484A, Q493R, Q498R, N501Y, Y505H, D614G, H655Y, N679K, P681H) and three amino acid deletions (L24-, P25- and P26-) were found in the S1 subunit of S protein and two amino acid substitutions (T223I and T269M) were found in ORF3a. ORF3a regulates the apoptosis and inflammatory responses in the infected cells. ORF3a can also activate the innate immune signaling receptor NLRP3 inflammasome, which results in tissue inflammation and cytokine production [22]. In addition to the above structural and accessory proteins, from our viral genome analysis, one amino acid substitution (P10S) and three amino acid deletions (E27-, N28- and A29-) were found in the ORF9b of Omicron variant. SARS-CoV-2 ORF9b can form a complex with a mitochondrial import receptor, Tom70. Recent studies have demonstrated that this complex can modulate the host immune response by compromising type I interferon synthesis [23,24]. However, whether these mutations in ORF9b can enhance the deleterious functions of ORF9b of Omicron variant to case infection in pediatric population, remains to be determined by further functional studies.

In this study, a novel frameshift mutation caused by insertion (14760T) was found at the NSP12 of the ORF1b region. NSP12 is a 932-amino acid long protein. It is an essential RNA-dependent RNA polymerase (RdRp) of the replication transcription complex (RTC) responsible for making copies of genomic and subgenomic RNAs and polymerizing antisense RNA during viral replication of SARS-CoV-2 [25]. During the replication of SARS-CoV-2 genome, NSP12 binds to its cofactors NSP7 and NSP8 to activate its capability to replicate long RNA [26]. NSP8 can initiate the replication process and operate as a primase [27,28]. NSP7 participates in the viral replication process by binding to NSP12 as another primase [27]. Therefore, NSP12-NSP7-NSP8 complex is one of the potential targets for the development of antiviral agents and COVID-19 therapies. However, no association between this frameshift mutation identified in this study and the transmission and severity of COVID-19 can be identified so far. Other than that, no novel mutations were found in the genome of the SARS-CoV-2 Omicron variant isolated from infected pediatric population. There was also no significant difference in the genomes of the SARS-CoV-2 Omicron variant infecting the pediatric population, compared to those of the infecting adults. Without any specific mutations in the genome of SARS-CoV-2 infecting pediatric population, these findings and the difference in the distribution of antibodies to structural proteins and accessory proteins of SARS-CoV-2 detected by Hachim et al. [16] support the hypothesis that SARS-CoV-2 Omicron variant may have different pathogenesis in pediatric population, when compared to adults and other variants. Another possible reason for the increasing number of infected pediatric population during Omicron variant-dominated outbreak may be the altered TMPRSS2 usage by SARS-CoV-2 Omicron variants [29,30]. One of reasons that children were less affected clinically by SARS-CoV-2 was due to lower ACE2 and TMPRSS2 expression compared to adults. However, with over thirty mutations in the S protein, the S protein of Omicron variant was found to be less efficiently cleaved compared to other variants such as Alpha and Delta [29]. The less efficient cleavage of S protein and defect in the entry of Omicron variant promotes the change in the use of TMPRSS2 for viral entry and hence altering the entry pathway of Omicron variant to the cathepsin-dependent endosomal route, which is TMPRSS2 independent [29]. A high ratio of C-to-T nucleotide mutations were found (40.7%) in the genome of Omicron variant. It could be the results of host-driven antiviral editing [31]. We have also identified a total of seven PCR primers and probes changes in WHO listed SARS-CoV-2 primers and probes in Omicron subvariants BA.2.2 and BA.2.10.1 in this study. C28770T locates within the N gene probe of Charité, G28881A, G28882A and G28883C locate within the N gene forward primer of China CDC, and C28311T locates within the N1 gene probe of US CDC. T15521A locates within the RdRp gene reverse primer of Charité. C26270T locates within the E gene forward primer of Charité. These mutations in the target regions of PCR primers and probes could result in false negative results during PCR detection. Nucleic acid amplification tests (NAAT) such as real-time reverse transcription polymerase chain reaction (rRT-PCR) are still the preferred method for SARS-CoV-2 detection worldwide. Therefore, it is suggested that dual targets are crucially important to minimize the risk of false negative results of detecting Omicron variant [32,33]. Considering the primer and probe binding site changes as the virus evolve, RT-PCR with dual target design is an important strategy to improve assay robustness to mutations in primer and probe binding sites. Some of the mutations in primer and probe sites may cause binding efficiency to drop hence could yield false negative results [34]. Examples of dual target like assays developed by Tombuloglu et al. targeting viral RdRP gene, viral E gene [32] or N gene [33] and human RNase P gene as internal control. NAAT such rRT-PCR assays are the standard method for SARS-CoV-2 detection worldwide, for their high sensitivity and specificity. Novel molecular assays were developed to detect SARS-CoV-2 viruses in the attempt to produce assays that do not rely on specialized molecular laboratory environment to perform. These assays improve accessibility of molecular tests in aid of better contact tracing efforts. Examples of such assays are CRISPR-Cas protein based, and reverse transcription loop-mediated isothermal amplification (RT-LAMP) methods. One-pot formats of these assays could be performed on a heat-block or water bath and be used in resource-poor situations. CRISPR-Cas9 methods first amplify SARS-CoV-2 RNA in samples with isothermal amplification, then detect by gRNA binding to SARS-CoV-2 genome and the Cas protein cleave reporter RNA strand to give a signal. CRISPR-Cas related methods could detect SARS-CoV-2 at 10–100 sequences per microliter of sample [35] and lateral flow strips to detect end point. RT-LAMP assays targeting N gene, RdRp gene, S and E genes were made with the possibility to use colorimetric detection with pH indicators [36], which could remove the need of fluorometric instruments. These novel methods could be further implemented in areas with less access to complex instruments.

From this study, we also found that fever (94.1%), cough (33.8%), running nose (33.8%), sore throat (32.4%) and vomiting (23.5%) were the more commonly reported symptoms in children infected by Omicron variant. For adults who were infected by Omicron variant, headache (76.5%), running nose (74.9%), sneezing (69.3%) and sore throat (68.4%) were the more commonly reported symptoms. Only 39.2% and 18.7% of the infected adults presented fever and diarrhoea respectively [37]. There is no evidence to show that children infected by Omicron subvariant BA.2.10.1 would result in worse and more severe clinical outcomes than children infected by BA.2.2 and no sexual dimorphism was observed. Our SARS-CoV-2 Omicron subvariants BA.2.2 and BA.2.10.1 genome sequences enriched the understanding of SARS-CoV-2 mutational landscape and improved the understanding of SARS-CoV-2 infection in pediatric population. The strengths of this study include a high average sequencing depth and a high average genomic coverage of the whole viral genome sequencing. Host-derived DNA sequences were removed to allow greater sequencing depth of the viral genome. The limitations of this study are that Sanger sequencing was not performed to confirm the mutations identified. Also, our cohort did not include any cases of the children with MIS-C.

### Conclusion

In conclusion, asymptomatic infection and transmission among children infected by Omicron subvariants BA.2.2 and BA.2.10.1 are not common. A novel frameshift mutation was found in the ORF1b region (NSP12) of the genome of Omicron variant in this study. There was also no significant difference between the genomes of the SARS-CoV-2 Omicron variant infecting the pediatric population, and those infecting the adults. However, Omicron variant may have different pathogenesis in pediatric population, when compared to adults.
